# Circulating miR-21 as a prognostic and predictive biomarker in oral squamous cell carcinoma

**DOI:** 10.12669/pjms.35.5.331

**Published:** 2019

**Authors:** Nosheen Mahmood, Muhammad Hanif, Akhtar Ahmed, Qamar Jamal, Shamim Mushtaq, Adnan Khan, Muhammad Saqib

**Affiliations:** 1 Dr. Nosheen Mahmood, MBBS, M.Phil, M.Phil Pathology, PhD Fellow. Department of Pathology, Ziauddin Medical University, Karachi, Pakistan; 2 Dr. Muhammad Hanif, PhD (Microbiology) Head of Department (Pr. Scientist) Clinical labs & Molecular Biology Lab, Karachi Institute of Radiotherapy and Nuclear Medicine (KIRAN), Karachi, Pakistan; 3 Dr. Akhtar Ahmed, FCPS. Karachi Institute of Radiotherapy and Nuclear Medicine (KIRAN), Karachi, Pakistan; 4 Dr. Qamar Jamal, PhD Pathology, Professor of Pathology, Ziauddin Medical University, Karachi, Pakistan; 5 Dr. Shamim Mushtaq, PhD. Associate Professor, Ziauddin Medical University, Karachi, Pakistan; 6 Dr. Adnan Khan, PhD. Karachi Institute of Radiotherapy and Nuclear Medicine (KIRAN), Karachi, Pakistan; 7 Mr. Muhammad Saqib, M.Sc. Karachi Institute of Radiotherapy and Nuclear Medicine (KIRAN), Karachi, Pakistan

**Keywords:** miRNA, Oral cancer, Real time PCR, miRNA-21

## Abstract

**Background & Objective:**

The high-throughput analysis of circulating microRNAs (miRNAs) is an active area of biomarker research. The oral cancer remains a common cancer among Pakistani males that continues to present at an advance stage, thus exhibiting poor survival. MiRNA 21 (miR-21) is the most consistently over-expressed miRNA in different types of tumor tissues. However, information regarding expression of miR-21 in plasma remains to be resolved. Therefore, present study was designed to investigate if miR-21 was expressed in plasma of patients with oral cancer, and further explore its diagnostic and prognostic potential.

**Methods:**

Present study was conducted at Ziauddin University and Karachi Institute of Radiotherapy and Nuclear Medicine (KIRAN). Histologically confirmed cases of oral squamous cell carcinoma were recruited from Oncology Department of Ziauddin Hospital between 2013 and 2017. Controls were carefully selected after considering age, gender and socioeconomic condition. MiRNA was extracted and immediately reverse transcribed to cDNA. MiR-21 expression was evaluated using probes specifically designed for Real time quantitative polymerase chain reaction.

**Results:**

A significant over expression of miRNA 21 was observed in histologically confirmed cases as compared to controls. The increased expression of miRNA 21 was also reported to be associated with tumor size, metastasis and local invasion (p<0.05).

**Conclusion:**

The expression of circulating miR-21 in plasma samples of oral cancer patients makes it a promising diagnostic and prognostic marker.

## INTRODUCTION

Oral cancer contributes to a significant proportion of cancer related morbidity and mortality in Pakistan.^1^ Treatment guidelines are being modified and updated at an escalating rate but survival outcome in advanced stage has failed to improve over several decades. Most tragic part is that majority of the patients are diagnosed at an advance stage.^2^ In this scenario the major focus of the research should be to identify markers which could predict prognosis and help in streamlining individualized treatment.

MicroRNAs can serve this purpose as recent researches have revealed their potential capability of being used as diagnostic, prognostic and predictive tool in cancers. Promising results has been the main driver of extensive research in the field of microRNAs. Therapeutic interventions that can be made to replace or suppress deregulated miRNAs have added further to the quest of exploring these molecular targets.^3^ Interesting enough miRNAs have not only been discovered in tumor tissue but can also be recovered from body fluids including serum and plasma of cancer patients and thus may serve as an important minimally invasive diagnostic tool.^4^

Circulating miRNAs are highly stable, owing to protection against RNAses offered by modifications like uridinylation, adenylation and methylations. Moreover, enclosure in micro vesicles and binding to RNA binding proteins contribute further to their stability in blood. This makes circulating miRNAs an attractive diagnostic tool.^5^

MiR-21 is one of the oncogenic microRNAs which promotes carcinogenesis via its anti apoptotic effect. MiRNA-21 gene is located at intron 10 on chromosome 17. Generally, miR-21 is observed to be up-regulated in cancers, and its suppression causes downsizing of tumor. It is also observed to contribute chemo resistance to the tumors.^6^ It also contributes in controlling genes like PTEN, TGF β, PDCD4 which are involved in the initial stage of cancers.^7^ Wei, 2011 and Yang Yu et al. experimentally validated PTEN as a target gene for miR-21. They found mir-21 inhibiting PTEN a tumor suppressor gene in prostate cancer cell line causing enhanced proliferation and invasion.^8^ It is proposed that miR-21 is involved in carcinogenesis via its ability to inhibit apoptosis.^9^ To validate this and to find out the effect of miR-21 on its target genes Ma X *et al*. performed experiments on mice. They knocked out miR-21 allele in mouse ES. To achieve this they generated a vector which replaces precursor to miR-21 with neomycin (NEO) –resistance expression cassette. They observed miR-21 deficient mice to have significantly lower rate of papilloma development suggesting a protective effect against chemically induced skin carcinogenesis.^10^

Gombos K et al. also concluded from their work that mir-21 has a higher expression in Oral cancer.^11^ Expression of miR-21 in Oral cancer tumor tissue has been researched, however utility of the same as a plasma biomarker remains unknown. Objective of this study was to investigate expression of miR-21 in plasma of Oral cancer patients, to explore its diagnostic potential and to correlate the expression with various clinicopathological variables of Oral cancer.

## METHODS

Present study was carried out at Ziauddin University and Karachi Institute of Radiotherapy and Nuclear Medicine (KIRAN). A total of 100 biopsy proven cases of Oral Squamous cell carcinoma were recruited via purposive sampling from Oncology department Ziauddin University between June 2013 and April 2017. Controls were carefully selected as healthy individuals of similar age group and gender as of tests. These controls were either healthy blood related family members of the cases or healthy individuals from same socio economic group. Board of Advanced studies and Ethical Review Committee of Ziauddin University gave approval of the protocol and written informed consent was obtained from all study subjects.

After conducting an interview and physical examination, 10 ml of venous blood was collected in EDTA tubes. Samples were left for 15 mins and the supernatant buffy coat plasma was transferred to Eppendorf tube for RNA extraction.

### RNA Extraction

RNA was extracted within 12-18 hrs of blood collection and cDNA synthesized immediately from eluted RNA to avoid any chances of RNA degradation. RNA was extracted through Favorgen Nucleic acid extraction kit. Total RNA was extracted according to kit’s protocol.

### cDNA synthesis

cDNA was synthesized via Thermo fisher Revert Aid cDNA Synthesis kit. For cDNA preparation we added MMLV RT 4 µl, dNTPs 2 µl, DTT 0.5 µl, Oligo dT primer 1µl, reverted RT 1 µl, RNAse inhibitor 1 µl, distilled H_2_O 3.25 µl and eluted miRNA 4 µl to prepare a final volume of 17 µl. cDNA was prepared according to the set protocol as follows, incubation at 42º C for 50 min, then 70ºC for 10 min and then 4ºC. The cDNA was then stored at -80ºC until further analysis.

### MiR-21 Expression

The same set of primers were used as previously reported by Wei et al. 12 Following set of primers were used for sequencing

5’UAGCAGCACGUAAAUAUUGGCG3’ for miR-16 5’ UAGCUUAUCAGACUGAUGUUGA3’ for miR-21; and 5’UCACCGGGUGUAAAUCAGCUUG3’ for cel-miR-39.

Considering low yield of miRNA in circulation its integrity and successful cDNA synthesis was evaluated by expression of GAPDH gene according to protocol by Thermo scientific. Samples which expressed a 496 kb band of GAPDH were further processed for miR-21 analysis. The amplification of internal control miR-16 was considered as a further proof of good quality miRNA extraction. For normalization of data mir-16 was used as endogenous controls.^12^

Real-time PCR was performed using SYBR green quantitative PCR reagent kit (Thermo scientific) on BioRad CFX96 analyzer. Each sample was analyzed in duplicate and appropriate negative controls were included. The 13 µl PCR volume for amplification included 2µl of cDNA, 6.5 µl of SYBR Green PCR Master Mix, 1.5 µl of primers and 3µl of H_2_O. The reaction mixture was run at 95°C for 3 min, followed by 40 cycles of 95°C for 5 s, 62°C for 35 s in which fluorescence was acquired. Rox was used as back ground noise on PCR program. Each sample was tested in duplicate. The expression levels of miR-21 were calculated utilizing the 2^–ΔΔ^ Ct method.^13^

### Statistical analysis

Statistical analysis of data was done via SPSS version 24. To express outcomes of qualitative variables frequency and percentages were used whereas mean with standard deviation was used for continuous variables. For comparison of variables chi square test was used for qualitative variables. Quantitative variables were compared using Students t test in sets of two groups and one way ANOVA for more than two groups. To differentiate miR-21 between cases and controls Receiver Operating Curves (ROC) were generated. Pearson’s correlation was used to correlate quantitative variables. A p value of < 0.05 was considered statistically significant.

## RESULTS

**Fig 1:**
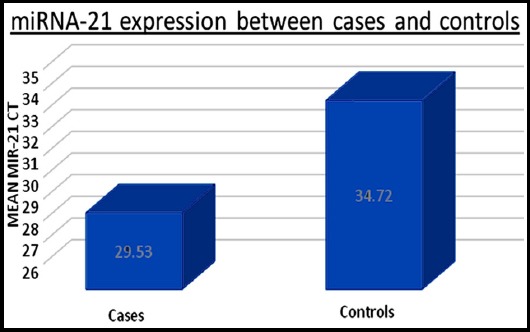
MiR- 21 is upregulated in Oral cancer as indicated by lower CT. MiR 21 expression in serum of Oral cancers and healthy controls determined by qRT-PCR (quantitative reverse transcription polymerase chain reaction. *p≤0.05.

A total of 100 samples (Oral cancer patients) and 100 controls were selected in the present study. Age and sex were not significantly different between cases and controls. There were 136 males and 64 females in both groups and average ct for both groups was same, 32.29 ± 4.98 for males vs. 31.77± 5.4 for females, p=0.507, CI (-1.0164 to -2.0510). miR-21 expression was significantly higher among cases as compared to controls 29.4 ± 5.3 ct vs. 34.7± 3.95 Ct, p<0.005 (Fig 1). The cutoff for ct was set at 35 cycles, any expression below 35 ct was marked negative. To calculate relative expression Δ CT or Livek method was used. Δ CT for cases was --5.39 whereas ΔCT for controls was -1.0371. ΔΔ CT of -6.4319 showed that cases had 6.3 times higher expression as compared to controls.

We did not observe any significant association between miR-21 expression and age, gender, ethnicity and smoking status. Out of 100 cases we had 67 Urdu speaking, 14 Sindi, 7 Baloch, 5 Punjabi, 3 Pashtoons and 4 others and expression of miR-21 was not different among various ethnic groups, p=0.869.

Upon comparing miR-21 level with tumor characteristics we did not observe any significant association with Tumor grade, nodal status and tumor recurrence. However, among different tumor sizes, a significantly higher expression was observed if tumor size was >4cm, p<0.001. Metastatic tumors and locally invasive tumors also showed a significantly higher expression of miR-21 as shown in Table-I. Hence we suggest that circulating miR-21 originates from tumor mass as suggested by a positive association with tumor size, local invasion and metastasis.

**Table I:** Relationship between Plasma miR 21 expression and Clinicopathological variables of Oral Cancer.

Characteristic		Number	Mean ± Standard Deviation	Confidence Interval	P-value
Age	< 40 yrs	36	29.91± 4.2	-1.402 to 2.621	0.549
	>40 yrs	64	29.30± 5.18		
Sex	Male	69	29.91± 4.87	-0.840 to 3.313	0.240
	Females	31	28.67±4.67		
Smoking status	Yes	66	29.98±4.7	-0.696 to 3.35	0.196
	No	34	28.65±4.94		
Grade	Well differentiated	14	29.5± 4.8		0.490
	Moderate differentiated	70	29.8±4.6		
	Poor differentiated	16	28.2± 5.6		
Tumor Size	< 4cm	50	31.37± 3.69	1.91-5.48	<0.001
	≥ 4 cm	50	27.67±5.1		
Nodal Status	Node negative	6	29.18± 4.67	-4.43 to 3.70	0.859
	Node Positive	94	29.5±4.8		
Metastasis	No	93	29.8±4.7	1.53 to 9.41	0.007
	Yes	6	24.4±4.42		
Local Invasion	No	81	30.06±4.54	0.4124 to 5.214	0.022
	Yes	19	27.2±5.5		

We had 7 patients who presented with tumor recurrence and they had a similar expression to the patients who presented with primary tumor, 30.57± 1.71 vs. 29.44 ± 5.0, p=0.557, CI (-2.66115-4.90919).

**Fig.2:**
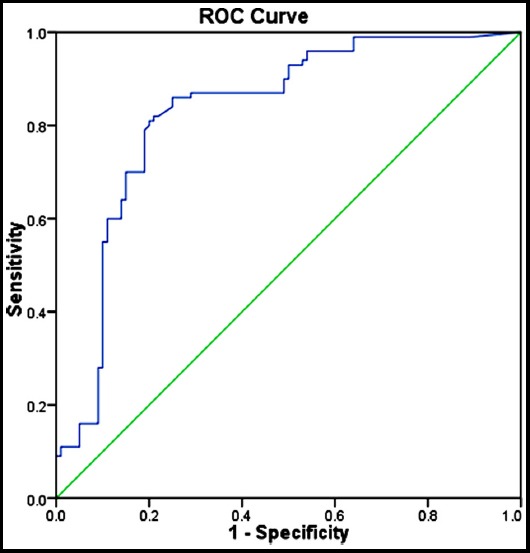
Receiver operating characteristic (ROC) curve analysis for miR-21 based on RT-qPCR data in discriminating oral cancer from controls. Area under curve 0.829, p<0.001.

To calculate sensitivity and specificity of the marker any expression seen after 35 Ct was considered as negative. We found miR-21 had a sensitivity of 91% and specificity of 54% Receiver operating characteristic (ROC) curve was generated based on ct values of miR-21. The Area under curve (AUC) was 0.829, p<0.001 suggesting that miR-21 can be used as a marker to discriminate Oral cancer from normal healthy control (Fig.2).

## DISCUSSION

A higher expression of miR-21 in patients with oral squamous cell carcinoma was observed. miR-21 is amongst the first miRNA observed to be expressed in serum. Arantes et al. investigated role of miRNAs in Head and neck cancer by extracting miRNA from tumor tissue. To start with, they screened 15 cases on a microarray to identify miRNAs deregulated in Oral cancer and found mir-21 to be over expressed. They validated their findings on a larger set of patients using miR-21 specific primers on real time PCR and found miR-21 to be an independent prognostic factor in a model adjusted for age, tumor site and tumor resectability.^14^ Similarly, Hedback N et al. investigated the expression of miR 21 in paraffin embedded tumor tissue and adjacent normal tissue from oral cancer cases using in situ hybridization. They observed a higher expression not only in cancer cells but also in tumor stroma and tumor blood vessels. Higher expression in tumor stroma was found to have a negative prognostic value on a multivariate analysis after adjusting for N stage and perineural invasion.^15^

Another study by Gombos et al. also checked for the miR-21 expression in tumor tissue and compared expression in the adjacent normal tissue. Upon comparing the expression through paired test they observed a significant over expression of miR-21 and receiver operating characteristic (ROC) curve showed a sensitivity and specificity of above 90%.^11^

Major research done in the field of miRNA in Oral cancers is either on the cell lines or surgically resected tumor specimens. One major disadvantage of a tissue based biomarker is invasiveness associated with the procedure. To overcome this identification of circulating biomarkers has gained considerable momentum. However, assuming the low concentration of miRNA in circulation very little has been done to search for circulating miRNAs in blood. Tachiban et al. used a high throughput highly sensitive array to determine circulating miRNA profile. On a profile of 1211 human miRNAs 20 showed more than two fold change compared to healthy controls. Of these 20, 16 were up regulated and 4 down regulated. Mir-223 showed the most significant up regulation so it was further validated on real time PCR and was proposed as a potential biomarker for diagnosis of oral cancer.^16^ Tachibana *et al* and Reis *et al* discovered miR-186, miR-3651 and miR- 494 to be deregulated in serum of OSCCA.^16^,^17^

We also report a higher expression of miR-21 but, interestingly we measured miR-21 in serum of OSCCA patients making it possible to chalk out patient’s prognosis via a less invasive serum biomarker. Shah S et al. conducted a comprehensive review of researches published on miR-21 in Oral squamous cell carcinoma and concluded a negative correlation with prognosis. In addition, they highlighted the therapeutic potential of inserting anticancer construct in miR-21 promotor region.^18^

Unlike breast, prostate, ovarian and liver cancer research has failed to discover a biomarker which could help in diagnosis and predict recurrence in oral cancer. The need is to dig deep in to the molecular events involved in oral cancer and to search for molecular biomarkers which could enable timely diagnosis and predict recurrence and metastasis.

### Limitations of this study

It is selection from a single center which is likely to contribute to selection bias and hence compromise on generalizability of results. Secondly we only compared expression of miR-21 between cases and controls, adding another group of patients with pre malignant Oral lesions would have served to add important information. Future multicenter studies with a larger sample size and premalignant patients would help in exploring its role as potential screening marker in oral cancer.

## CONCLUSION

Our findings suggest that serum miR-21 levels reflect tissue mir21 expression and hence it may be used as a non invasive tool for predicting tumor invasion and metastasis.

### Author`s Contribution

**NM and MH** did collection, designed, manuscript writing, and editing of manuscript.

**MS, SM and AK** did data collection, sample run, analysis, and editing of manuscript.

**QJ and AH** did review, editing and final approval of manuscript.
